# Technostress Creators and Job Performance Among Frontliners: Theorizing the Moderating Role of Self-Efficacy

**DOI:** 10.3389/fpsyg.2022.827027

**Published:** 2022-06-29

**Authors:** Jeannette Saidy, Zanete Garanti, Richard Sadaka

**Affiliations:** ^1^Department of Business Administration, Cyprus International University, Lefkosa, Turkey; ^2^Department of Business, City Unity College Nicosia, Nicosia, Cyprus; ^3^Department of Economics and Business Administration, Lebanese University, Beirut, Lebanon

**Keywords:** technostress, self-efficacy, performance, transactional model of stress and coping, frontline employees

## Abstract

Technostress is evolving as an imperative area of academic research amid the “new normal” settings of working remotely. Research has investigated the relationships between technostress and job outcomes and proposed individual- and organizational-level approaches to manage it. However, insights into the influence of dynamic personality differences on this relationship are limited. This study ties the concept of self-efficacy to the transactional model of stress and coping, and investigates to what extent computer and social self-efficacy moderate the relationships between technostress creators and frontline employee’s job performance. Findings shift the focus from the negative aspects of technostress and outcomes to both positive and negative aspects. This study’s contributions and implications for theory and practice are discussed.

## Introduction

Technology has permeated individuals’ personal and professional lives. Nowadays, society is living in organized chaos controlled by technology’s ever-evolving demands and repercussions. Since the global health crisis started in 2019, most of the world’s population is working, studying, and meeting online, and technology is becoming an ever more prominent aspect of people’s lives. Humans create and shape technologies; however, these technologies then shape their lives for worse or better, depending on what these technologies enable or hamper along with people’s prudence in allowing the machine to take over.

This dilemma sheds light on a rich organizational research topic related to both stress and technology. Brod (1984) created the term technostress to mean “a modern disease caused by one’s inability to cope or deal with technology in a healthy manner” (p. 16). Later, with the propagation of information and communication technology (ICT) use in different organizations, this definition was extended to include “the stress caused by an individual’s attempts to deal with constantly evolving ICTs and the changing physical, social, and cognitive responses demanded by their use” (Hwang and Cha, 2018, p. 283).

Using ICT has become a universal impetus in organizational settings, resulting in a significant gain in innovations and process efficiencies. However, dealing daily with technology generates different technostress creators (TSCs) that may promote employee technostress (Tarafdar et al., 2011), which is generally associated with adverse job-related outcomes (Jena, 2015). How to reduce ICT-related exposure is a recent research topic and understanding the sources of undesirable behavioral and psychological reactions toward using ICT is important to be able to set necessary interventions that reduce the ICT exposure of ICT users (Busse et al., 2022).

Scholars have noted that the inconsistency of empirical research findings regarding stress results from failing to highlight the importance of individuals’ differences (Galluch et al., 2015). Recent technostress literature indicates a noticeable gap in the theoretical understanding of the impact of TSCs on job outcomes, particularly when considering individuals’ differences (Srivastava et al., 2015). The need for a more in-depth theoretical approach to this impact was emphasized in previous research (Tarafdar et al., 2015) since personality differences influence the choice of coping mechanisms in response to stressful experiences and play an explanatory role in psychological outcomes (Srivastava et al., 2015).

It has been hypothesized that stress is the proximal originator of job outcomes (Karatepe et al., 2018). Most technostress studies have tested models that posit a direct relationship between TSCs and negative job outcomes. This shortcoming led researchers to recently conceptualize individual- and organizational-specific approaches that counterbalance the causes and effects of TSC. Organizational approaches are reflected in various inhibitor mechanisms, including technology support, literacy facilitation, and technology involvement (Jena, 2015). Individual approaches are mostly reflected in various individual static characteristics, such as big five personality traits (Khedhaouria and Cucchi, 2019) and dynamic characteristics such as regulatory focus (Hwang and Cha, 2018).

Although previous research has enriched the literature, ICT is continuously evolving; it requires a user’s ability to adapt regularly to change (Tarafdar et al., 2011) and elucidate the critical roles that dynamic personality differences play in coping with technostress.

Self-efficacy (SE) is a dynamic personality construct that refers to the extent to which individuals believe in their abilities to perform what is required to meet a task’s demands (Bandura, 1977). SE is perceived as a resource to avoid negative strain consequences, facilitate employees’ adaptation to organizational changes (Shoji et al., 2016), and determine several stress-related outcomes (Bandura, 1997). Within the context of continuous technological changes, SE is considered the most convenient personal domain for defining the job outcomes influenced by technology (Krishnan, 2017). Scant research within the ICT context has tested the direct effect of SE on TSC (Tarafdar et al., 2011) and SE’s direct effect on job outcomes (Beas and Salanova, 2006). However, studies about SE’s outstanding role in countering technostress causes and outcomes are scarce.

Some research has suggested that work stressors lead to negative outcomes, but other research has failed to show this. The inconsistency in these findings has led researchers on occupational stress to admit that workplace stressors might lead to negative or positive outcomes depending on how these stressors are appraised and then managed (LePine et al., 2005). However, this concept has not been broadly tested (Webster et al., 2011).

Most researchers have conceptualized five TSCs as one construct and assumed that they have an identical nature and lead to identical outcomes. However, empirical and theoretical evidence (Tu et al., 2005; Ayyagari et al., 2011) contradict such an assumption. An individual’s behavior changes from situation to situation according to his or her perception, not because of the situation *per se* (Jones, 1989).

Stress is considered a context-specific phenomenon and includes specific roles, tasks, or technologies (Tarafdar et al., 2015). Frontline employees (FLEs) are often the main contact clientele have with an organization. In the field of digitization, FLEs play the main role in driving successful customer-service encounters even when technology complements the employee–client relationship. However, empirical research has rarely focused on the technostress aspect of FLE. Thus, the FLE context represents a major domain to explore the technostress phenomenon (Christ-Brendemühl and Schaarschmidt, 2020).

Following this line of thought, this study aims to fill the above-mentioned research gaps by theorizing a technostress appraisal and coping model for an FLE in an ICT context in a way that points to SE beliefs as possible interventions that have the potential to influence FLE’s perception of his ability to exercise control over each TSC and its consequences on work outcomes.

This study’s context is FLEs within ICT organizations in Lebanon who are exposed daily to technostress. Nowadays, Lebanon’s ICT sector, known as the economy’s key driver, is emerging as a global leader, exporting software engineering and services in the region. This industry’s innovation and performance are driven by ICT companies’ attempts to invest more in the labor force and customers’ requirements, necessitating successful customer relationship management (Ben Hassen, 2018).

## Theoretical Background and Literature Review

### Transactional Model of Stress and Coping

This study is grounded in the transactional model of stress and coping (TMSC) (Lazarus and Folkman, 1984). The model is concerned with change and process, and adequately considers the role of personality in generating possible positive responses to stress compared to most applied stress theories (LePine et al., 2005). Therefore, the TMSC can be aligned with two significant defies in this study: the continually evolving nature of ICT and the dynamic nature of self-efficacy construct.

The TMSC model points to the relationship between people and the environment as transactional, dynamic, bidirectional, and mutually reciprocal. These transactions rely heavily on the stressors’ effects, which are the demands of an internal or external environment that disturb the equilibrium of an individual’s psychological and physical well-being and necessitate action to restore balance. When faced with a stressor, the TMSC suggests that individuals cope with such disruptions by using two processes that unceasingly affect each other. Individuals promote a primary appraisal, which is a judgment about the weight of a stressor, and then promote a secondary appraisal to evaluate coping choices, and subsequently, perform diverse efforts to manage the situation which are the coping efforts.

The TMSC accounts for coping with disruptions and is applied in this study within ICT settings. Introducing and using innovative and complex ICTs in an organization is a disruptive event that engenders predictable and unpredictable consequences in an employee’s environment. They are construed differently by different individuals with various dominant personality characteristics prompting complex and varied individuals’ responses (Griffith, 1999). Accordingly, individuals with diverse personality characteristics will assess disruptive events triggered by ICTs differently, due to their varying external and internal demands.

Besides, from a management perspective, disruptive events are assessed as one of two key categories: either as an opportunity to improve employee’s job performance or as a threat to the job (Beaudry and Pinsonneault, 2005). Lazarus and Folkman (1984) identified disruptive conditions as multifaceted, emerging from both opportunities and threats.

In this study, SE is theorized as a primary form of coping. In this view, Lazarus and Folkman (1987) noted that “Situational appraisals of control, which are about actual encounters, vary with the circumstances, and Bandura’s concept of SE belongs in this category” (p. 148). The ability to cope with demands is the core of the SE theory associated with the TMSC. The latter also embraces assessing individuals’ capacity in matching demands along with coping resources, so the TMSC and SE’s links become obvious. More self-efficacious individuals are less likely to believe that work stressors tax their coping mechanisms; accordingly, they are less likely to experience strain.

Research conducted on different disciplines has defined the multifaceted character of task-specific SE in terms of its antecedents, outcomes, and moderating factors (Marakas et al., 1998). Consistent with this logic, this research posits that each type of TSC is a disruptive condition (Srivastava et al., 2015) that tends to be multifaceted and capable of being viewed as a threat or an opportunity and could lead to both positive and negative outcomes, depending on an individual’s SE beliefs. Theorizing and testing the impact of each TSC on individuals with different self-efficacy beliefs with respect to possible negative and positive outcomes constitutes a noteworthy contribution to the technostress literature in particular and the occupational stress literature in general.

### Technostress

Technostress creators are organizational stressors associated with the inefficient use of ICT that engender stress within a person. Ayyagari et al. (2011) acknowledged five categories of TSC: (1) work–home conflict (WHC), which describes conditions in which ICT use may create conflicts between the demands of work and home responsibilities; (2) invasion of privacy (IP) describes conditions in which ICT use may violate privacy by increasing surveillance; (3) work overload (WO) describes conditions in which ICT use may engender a workload that exceeds an individual’s skill level or capability; (4) role ambiguity (RA) describes conditions in which ICT use may induce a lack of information necessary to perform a role; and (5) job insecurity (JI) which describes conditions in which ICT invasion may lead someone to perceive a threat about losing his or her job.

Studies have examined technostress in government administrations (Fuglseth and Sørebø, 2014), in different industries (Ayyagari et al., 2011), among academicians (Jena, 2015), and social media (Maier et al., 2015). Studies have found that technostress harms employees’ health, results in job burnout (Khedhaouria and Cucchi, 2019), decreased employee performance, decreased job satisfaction, and lower organizational commitment (Hwang and Cha, 2018).

### Frontline Employees

Today, service systems embrace interrelated technologies, human actors, and processes (Larivière et al., 2017). In addition, technology might act as an augmenting force to FLEs’ and customers’ interaction in a service encounter context (De Keyser et al., 2019). Within this spectrum, this study focuses on technologies operated by FLEs either to support their core tasks or to counterpart encounters with the clienteles.

Organizational frontlines in the technology services industry are essential to business success. They can benefit from ICT as a means to provide services more accurately, rapidly, and with higher quality (Toivonen, 2016). Nevertheless, technological systems raise complexity, leading FLE to face additional, challenging demands, such as coping with modern equipment and addressing increasing performance standards (Subramony et al., 2017), generating FLE’s technostress.

### Self-Efficacy

Self-efficacy is a dynamic personality construct derived from social cognitive theory and originates from four sources: performance accomplishments, physiological and emotional arousal, social persuasion, and vicarious experience (Bandura, 1977). Bandura (1997) explained that: “efficacy beliefs should be measured in terms of particularized judgments of capability that may vary across realms of activity” (p. 42). Accordingly, the study focuses on two self-efficacy measures contingent on FLE’s task.

Computer self-efficacy (CSE) refers to the extent to which an individual believes he or she can utilize technology to succeed at a particular task (Compeau and Higgins, 1995), while social self-efficacy (SSE) is an individual’s perceived confidence and skills in a social situation (Grieve et al., 2014).

Computer self-efficacy is a vital construct in IS research (Gupta and Bostrom, 2019). However, people who frequently use technology internalize an aspiration to conquer the system more than developing human relationships and experiencing enjoyment (Brod, 1984). Among the various research concerning different social effectiveness constructs, SSE has been recognized as particularly important for the dimensions of an individual’s career success that necessitate social interaction (Luo et al., 2019).

### Job Outcomes

An information technology success can be measured through its impact on work at the users’ level (Michel et al., 2019), and users’ outcomes can be dichotomized into psychological and behavioral outcomes (Yavas and Babakus, 2010). This study is concerned with user’s behavioral outcomes illustrated by employee performance.

Job performance is a multidimensional concept of behavior necessary for organizations to achieve their strategic objectives and activities (Jena, 2015). In-role performance (IRP) refers to the formal duties and activities integrated into an employee’s job description. Extra-role performance (ERP) refers to work-related activities that go beyond the requirements stated in the job description (Caesens and Stinglhamber, 2014).

When managers evaluate an individual’s performance, in-role and extra-role performances significantly affect the organization’s overall performance (MacKenzie et al., 1998). They are also important in transferring the effects of the organization’s service climate into customer satisfaction and subsequently into the organization’s performance (Yavas and Babakus, 2010).

## Hypotheses Development

### Technostress Creators and Job Performance

Job stress literature admits that stress manifests in and is weighed through related workplace responses such as exhaustion, job dissatisfaction, burnout, absenteeism, reduced job performance (Tarafdar et al., 2015), and reduced organizational commitment (Lopopolo, 2002; Fox and Dale, 2008).

Job performance is a worthy key manifestation of the organization and an imperative variable for research. Emphasizing the significance and the aspects of performance in an organization can affect the usage of organizational resources for improved performance (Lavanson, 2007). Today, technology has become an “organizational actor,” not only a resource but working with constantly changing technology can improve or can diminish the performance. As far as technostress is concerned, studies show that Technostress negatively affects employee performance (Tarafdar et al., 2007, 2011, 2015).

From this limited examination of performance impacts of technostress, we note that each TSC can have adverse impacts on in-role or extra-role performance. The following hypotheses are proposed:

1. Technostress creator WHC (H1a), IP (H2a), WO (H3a), RA (H4a), and JI (H5a) will be negatively related to in-role performance.
2. Technostress creator WHC (H1b), IP (H2b), WO (H3b), RA (H4b), and JI (H5b) will be negatively related to extra-role performance.

### Social Self-Efficacy, Computer Self-Efficacy, and Job Performance

Self-efficacy is consistently the strongest indicator of intent among other evaluation measures of perceived behavioral control (Fu et al., 2010). Empirical research findings demonstrate a positive correlation between SE and an employee’s performance in different organizational settings (Carter et al., 2018). However, there are still some questions about SE’s capacity to influence performance within a complicated FLE’s job settings (Krishnan et al., 2013) and the literature lacks studies about the influence of domain-linked SE on employee performance.

Social self-efficacy has been conceptualized as an essential antecedent of job performance’s social component (Fan et al., 2013). Personnel with high SSE beliefs yield generally positive outcomes within social interaction tasks (Luo et al., 2019). Nonetheless, research about the effect of individual’s SSE in the workplace are scarce, given that most of them have been conducted on children’s and adolescent’s SSE. Fan et al. (2013) extended the SSE construct to the workplace settings and confirmed that it predicted political skills, organizational citizenship behavior, organizational self-esteem, and affective well-being. Later on, Luo et al. (2019) moved beyond Fan et al.’s (2013) study and empirically established the variable social status as a mediator between SSE and employee outcomes, namely, job satisfaction and peer-rated task performance.

Computer self-efficacy has been conceptualized as a strong predictor of diverse computing attitudes and beliefs (Hong et al., 2016) associated with more computer usage, increased performance, and reduced computer usage anxiety (Compeau and Higgins, 1995). Furthermore, competent technology users work with information systems in a smarter manner that is likely to enhance their ability to generate benefits from it for their tasks (Tarafdar et al., 2015).

On the basis of the abovementioned arguments, it is believed that CSE and SSE are likely to result in enhanced in-role and extra-role performances. Thus, the following hypotheses are proposed:

1. H6a: SSE will be positively related to IRP
2. H6b: SSE will be positively related to ERP
3. H7a: CSE will be positively related to IRP
4. H7b: CSE will be positively related to ERP

### Social Self-Efficacy as a Moderator Between Technostress Creator and Performance

This study suggests the construct of self-efficacy as a form of cognitive coping that moderates the relations between TSCs and job outcomes. SE engenders positive thoughts that impact stress by allowing positive interpretation of stressful situations. SE functions as a cognitive mechanism by which the person responds to stress with a sense of controllability (Bandura, 1997) and provides insight that effort will contribute to successful outcomes (Carter et al., 2018).

The infusion of technology into the personnel-intense service context has the potential to boost FLE’s performance by supporting them and enabling them to focus on main activities such as personal interactions with the clientele (Larivière et al., 2017). Nonetheless, using complex technologies might lead FLEs to face supplementary challenging task demands. For instance, confronting a system or a digital breakdown while collecting and storing clientele’s data might interrupt FLE’s work and cause him/her to lose time. Supposing that technology operates appropriately, it will necessitate FLEs’ attentiveness, which might distract him/her from rapport-building behaviors such as smiling or eye contact (Christ-Brendemühl and Schaarschmidt, 2020). SSE has been identified as the main construct in social interactions and is broadly used to explain an individual’s social behavior (Fan et al., 2013). Nevertheless, to the best of our knowledge, no study examined the prominent moderating role of SSE within the organizational technostress literature.

Individuals with high SSE tend to adopt proactive strategies during their social interactions (Gu et al., 2014) and encounter social activities’ difficulties with more resilience than individuals with low SSE counterparts (Meng et al., 2015). Consequently, they can bounce back and adjust to a new situation faster than others. Such positive attributes may generate advanced levels of achievement (Luo et al., 2019).

Given the increasing implementation of digital technologies that are transforming the nature of ICT services, and given the increasing task-related social interaction demands placed on FLEs in the ICT services industry, this study, based on the TMSC perspective, suggests that SSE may help FLEs maintain positive thoughts about their perceived ability to successfully participate in job-related social interactions influencing their performance despite the presence of task-related technological stressors. Thus, anticipating positive performance arising from SSE beliefs may counter anticipating such negative outcomes. Consequently, the threat of low performance based on facing any task-related TSC would be weaker or less probably to arise. In so doing, SSE assumes the moderator role, thus the following hypotheses are proposed:

1. Social self-efficacy moderates the relationships between work–home conflict (H1c), invasion of privacy (H2c), work overload (H3c), role ambiguity (H4c), and job insecurity (H5c) and individual in-role performance.
2. Social self-efficacy moderates the relationships between work–home conflict (H1d), invasion of privacy (H2d), work overload (H3d), role ambiguity (H4d), and job insecurity (H5d) and individual extra-role performance.

### Computer Self-Efficacy as a Moderator Between Technostress Creator and Performance

Computer self-efficacy constitutes a significant cognitive-estimative variable to measure an individual’s computer skills. Someone with high CSE contributes to solving difficulties triggered by computer technology and expresses a low level of technostress, while someone with low CSE engenders a negative perception of this technology (Dong et al., 2020).

Studies regarding inhibiting mechanisms on technology-enabled performance, particularly CSE, are nascent. The sales literature comprises evidence of the effect of mechanisms (e.g., user support, training) that would moderate the negative perception of technology on performance through an enhanced feeling of technology self-efficacy (Speier and Venkatesh, 2002; Geiger and Turley, 2006). Moreover, ICT self-efficacy has been found to play a shock-absorbing role against the anxiety related to ICT (Henderson et al., 1995) in a sense that it can decrease the negative impact of ICT usage leading to techno strain (Salanova et al., 2013) and can counter the increase in role stress and the decrease in performance that are due to technostress (Tarafdar et al., 2015).

Extending this reasoning and these findings to the FLE’s use of ICT, this research suggests that when FLEs face relevant excessive information processing and task demands within the ICT context, their CSE beliefs may help them maintain positive thoughts about their capacity to successfully control and perform computer-related tasks. Anticipating positive outcomes emerging from CSE may help to counter anticipating such negative outcomes, namely, job performance. So, the threat of low performance based on TSCs should be weaker and less likely to take place. The subsequent hypotheses reflect the probable role of CSE in countering the effects of TSCs and their influence on job performance:

1. Computer self-efficacy moderates the relationships between work–home conflict (H1e), invasion of privacy (H2e), work overload (H3e), role ambiguity (H4e), and job insecurity (H5e) and individual in-role performance.
2. Computer self-efficacy moderates the relationships between work–home conflict (H1f), invasion of privacy (H2f), work overload (H3f), role ambiguity (H4f), and job insecurity (H5f) and individual ERP.

The proposed relationships tested in this study are illustrated in the below conceptual model.

## Methodology

SPSS 26 was used to perform descriptive analysis on participants’ demographic characteristics and correlations among all the variables of this study. SMART-PLS3 was also used to run confirmatory factor analysis (CFA) to test the construct validity of all the measurements. Given that 380 participants provided data at the employee level (i.e., WHC, IP, WO, RA, JI, SSE, and CSE) and 57 participants provided data at the supervisor level [i.e., in-role performance (IRP) and extra-role performance (ERP)], our hypothesis testing necessitated hierarchical or cross-level techniques. Since linear regression modeling can resolve no independence difficulties and estimate the influences of factors at multiple levels simultaneously (Raudenbush and Bryk, 2002), hierarchical linear modeling (HLM) has been used as an analytic tool to test the causal and moderation hypotheses.

### Measurement Instrument

Technostress creator was measured using validated items from Ayyagari (2007), with five subscales with a 7-point Likert scale ranging from “strongly disagree (1)” to “strongly agree (7).” CSE was measured with 10 items on a 10-point Likert scale, developed by Compeau and Higgins (1995), ranging from “not at all confident (1)” to “totally confident (10).” SSE was measured with three items on a 7-point Likert scale, developed by Grieve et al. (2014), ranging from “strongly disagree (1)” to “strongly agree (7).” Answering the CSE measurement items required the respondents to imagine they have been given new software to achieve their tasks and answering SSE measurement items required respondents to imagine their ability in an interpersonal exchange. The outcome variables IRP and ERP were assessed directly by the FLE’s supervisor and measured using three items, each on a 7-point response scale, developed by Netemeyer and Maxham (2007), ranging from 7 (always) to 1 (never). Appendix A describes the questionnaire’s items and their codification. The questionnaires were prepared in English and then translated into Arabic using the back-translation method (McGorry, 2000). A pilot study using 2 samples of 10 employees and their direct supervisors indicated that no revision was necessary.

### Data Collection

The ICT services and manufacturing segment in Lebanon are estimated to employ approximately 10,700 persons, the majority of whom are skilled professionals (IDAL, 2019). Respondents were FLEs in ICT organizations in Lebanon and their direct supervisors. The data were collected during the first semester of 2021. The management staff of 22 large organizations were contacted *via* a letter to obtain consent for data collection, and 16 organizations with 57 supervisors opted to participate in this research. A list was prepared that comprised the respondents’ names, with an identification number assigned for each employee, which appeared on each employee’s and supervisor’s questionnaire. The questionnaires were matched *via* the identification number. A total of 400 questionnaires were distributed, and an adequate sample size of 380 questionnaires were collected. The organizations’ managerial support contributed to a 95% response rate. The questionnaires were answered by a self-administered method and sealed in an envelope to respect the respondent’s anonymity and privacy. The employees’ questionnaire included items about their demographic profile and measures of the five TSCs, CSE, and SSE.

Data about each FLE’s in-role and extra-role performances were assessed by the direct supervisor since using multiple informants assessments would help reduce the common-method variance (Yavas and Babakus, 2010).

### Demographics

The sample consisted of 246 (65%) male and 134 (35%) female respondents. The majority of respondents (45%) were 26–35 years old, 22% were 18–25 years old, and 33% were older than 35 years. About marital status: most respondents (55%) were single, 41% were married, and 4% were divorced. Regarding education: 6% of respondents have a primary school education, 48% have a bachelor’s degree, 42% have a master’s degree, and 4% have a doctoral degree. Regarding work experience: 32% had less than 5 years of experience, 33% had experience ranging from 5 to 10 years, 19% had experience ranging from 11 to 15 years and 16% had more than 15 years of experience. In terms of annual income, 40% of the surveyed samples earned less than 24,000 USD annually, 34% earned between 24,000 USD and 48,000 USD, 22% earned between 48,000 USD and 72,000 USD, and 4% earned over 72,000 USD (Appendix B).

## Results and Analysis

### Reliability and Validity Analysis

The CFA is assessed by two main components, convergence validity and discriminant validity. Table 1 represents the results of convergent validity which refers to the degree to numerous attempts to measure the same concept in agreement (Hair et al., 2010).

**TABLE 1:** Convergent validity and internal reliability.

Construct	Item	Factor loading	Average variance extracted (AVE)	Composite reliability (CR)	Internal reliability Cronbach alpha
**Employee level (*n* = 380)**					
Work-home conflict (WHC)	WHC1	0.953	0.920	0.972	0.956
	WHC2	0.967			
	WHC3	0.957			
Invasion of privacy (IP)	IP1	0.894	0.882	0.968	0.955
	IP2	0.959			
	IP3	0.961			
	IP4	0.941			
Work overload (WO)	WO1	0.927	0.902	0.965	0.945
	WO2	0.964			
	WO3	0.958			
Role ambiguity (RA)	RA1	0.942	0.892	0.970	0.959
	RA2	0.942			
	RA3	0.953			
	RA4	0.940			
Job insecurity (JI)	JI1	0.951	0.905	0.966	0.947
	JI2	0.962			
	JI3	0.941			
Social self-efficacy (SSE)	SSE1	0.943	0.927	0.974	0.961
	SSE2	0.977			
	SSE3	0.968			
Computer self-efficacy (CSE)	CSE1	0.913	0.852	0.983	0.981
	CSE2	0.917			
	CSE3	0.919			
	CSE4	0.926			
	CSE5	0.919			
	CSE6	0.924			
	CSE7	0.928			
	CSE8	0.918			
	CSE9	0.927			
	CSE10	0.936			
Supervisor level (*n* = 57)					
In-role performance (IRP)	IRP1	0.947	0.924	0.973	0.959
	IRP2	0.974			
	IRP3	0.963			
Extra-role performance (ERP)	ERP1	0.977	0.966	0.988	0.983
	ERP2	0.987			
	ERP3	0.985			

As shown in Table 1, the results of assessing the standardized loadings of the items showed that the factor loading of all 36 items was more than 0.5 as recommended by Hair et al. (2006), which ranged between 0.894 (for IP1) and 0.987 (for ERP2). The average variance extracted (AVE) of all the variables was above 0.5, ranging between 0.852 (for CSE) and 0.966 [for extra-role performance (ERP)]. The composite reliability (CR) ranged between 0.965 (for WO) and 0.988 [for extra-role performance (ERP)], which was higher than the suggested value of 0.6 (Hair et al., 2010). The values of Cronbach alpha were more than 0.7 as recommended by Nunnally and Bernstein (1994), ranged between 0.945 (for WO) and 0.983 [for extra-role performance (ERP)]. These results indicate a satisfactory convergent validity.

Table 2 presents the scale, means, and standard deviations of the constructs. Discriminant validity, which refers to the issue of how truly distinct a construct is from other constructs, is also presented in Table 2, using two approaches: (1) Fornell and Larcker (1981) approach to compare the standardized correlations and square root of AVE. (2) Henseler et al. (2015) approach to evaluate the results of heterotrait–monotrait ratio of correlations (HTMT).

**TABLE 2:** Descriptive statistics and discriminant validity, using Fornell and Larcker approaches and HTMT.

	LS	Mean	SD	CSE	ERP	IP	IRP	JI	RA	SSE	WHC	WO
CSE	10	6.399	2.295	**0.923**	0.756	0.432	0.860	0.748	0.732	0.837	0.550	0.748
ERP	6	3.893	2.093	0.743	**0.983**	0.322	0.867	0.690	0.666	0.762	0.466	0.590
IP	7	4.976	1.591	–0.420	–0.313	**0.939**	0.368	0.373	0.326	0.444	0.319	0.409
IRP	6	5.476	1.507	0.835	0.842	–0.355	**0.961**	0.751	0.718	0.864	0.562	0.717
JI	7	3.458	1.854	–0.721	–0.666	0.356	–0.718	**0.951**	0.791	0.794	0.532	0.656
RA	7	3.312	1.586	–0.710	–0.646	0.314	–0.691	0.754	**0.944**	0.803	0.482	0.660
SSE	7	4.975	1.594	0.814	0.741	–0.428	0.831	–0.758	–0.771	**0.963**	0.557	0.732
WHC	7	2.940	1.770	–0.533	–0.452	0.304	–0.540	0.506	0.462	–0.533	**0.959**	0.470
WO	7	3.849	1.669	–0.720	–0.569	0.389	–0.683	0.621	0.628	–0.698	0.447	**0.950**

*N = 57 (supervisors); N = 380 (employees); bolded values on the diagonal display the square root of the average variance extracted; values below the diagonal display standardized correlations; values above the diagonal display HTMT results. CSE, computer self efficacy; ERP, extra-role performance; IP, invasion of privacy; IRP, in-role performance; JI, job insecurity; RA, role ambiguity; SSE, social self efficacy; WHC, work-home conflict; WO, work overload; SD, standard deviation; LS, Likert scale.*

As shown in Table 2, the square root of the average variance extracted for each construct is higher than the correlations of that construct with other constructs (Hair et al., 2010). Furthermore, the correlations between constructs were all less than the threshold 0.85, ranging between −0.771 (correlation between RA and SSE) and 0.842 (correlation between IRP and ERP), indicating a satisfactory discriminant validity between the constructs (Kline, 2010). The HTMT values of the latent constructs were below 0.90, and ranged between 0.319 (between IP and WHC) and 0.867 (between IRP and ERP). Therefore, it confirms that each latent construct measurement was discriminating from each other (Henseler et al., 2015).

Table 2 also represents the descriptive statistics of the constructs, including the Likert-point scale, mean, and standard deviation.

The result indicated a good model fit. The SRMR was 0.031, below the threshold of 0.08 as recommended by Hu and Bentler (1999). The NFI was 0.903, above the threshold of 0.9, representing an acceptable fit (Hair et al., 2006; Ho, 2006).

### Hypotheses Findings – Hierarchical Linear Modeling

In the HLM analyses, a fully unconditional, intercept-only model for IRP and ERP was first estimated to examine supervisor within-group and between-group variability. Significant within-group variances in supervisor were found (IRP: σ^2^ = 2.032, *p* < 0.001; ERP: σ^2^ = 3.880, *p* < 0.001). Significant between-group variances in supervisor were found (IRP: τ = 0.239, *p* < 0.05; ERP: τ = 0.499, *p* < 0.05).

Intra-class correlation coefficient (ICC) for IRP and ERP was 0.105 and 0.114, respectively, above the threshold of 0.05 (Heck et al., 2014). In other words, 10.5% of the total variation IRP and 11.4% of the total variation in ERP occurs between supervisor groups. The significance between and within-group variances indicates that there may be supervisor-related factors that help to explain variation between supervisors in IRP and ERP.

In other words, these variances demonstrate the nested nature of data and justified our use of multilevel analyses. The differences of Chi-square tests with deviance values indicated that Model 2 represented a significantly better fit than Model 1 [IRP: Δχ^2^(1) = −405.564, *p* < 0.001; ERP: Δχ^2^(1) = −281.750, *p* < 0.001], Model 3 had a better fit than Model 2 [IRP: Δχ^2^(1) = −169.663, *p* < 0.001; ERP: Δχ^2^(1) = −92.119, *p* < 0.001], and Model 4 had a better fit than Model 3 [IRP: Δχ^2^(1) = −25.464, *p* < 0.001; ERP: Δχ^2^(1) = −15.172, *p* < 0.001]. The Pseudo *R*^2^ values supported the validity of all models. Table 3 presents the results of examining causal and moderation hypotheses, using HLM.

**TABLE 3:** Results of causal and moderation analysis, using HLM.

Predictor	In-role performance (IRP)				Extra-role performance (ERP)			
	**Model 1**	**Model 2**	**Model 3**	**Model 4**	**Model 1**	**Model 2**	**Model 3**	**Model 4**
No variable								
Intercept	5.464*** (0.099)	8.468*** (0.164)	2.389 ** (0.438)	4.626*** (0.907)	3.885*** (0.140)	7.483*** (0.268)	0.087 (0.789)	3.339* (1.647)
Independent variables								
WHC		−0.125*** ^*H*1.a^ (0.030)	−0.053* (0.025)	−0.016 (0.079)		−0.105* ^*H*1.b^ (0.049)	−0.012 (0.044)	−0.082 (0.143)
IP		−0.035 ^*H*2.a^ (0.031)	0.045 (0.026)	−0.156 (0.140)		**−0.049 ^*H*2.b^ (0.051)**	0.048 (0.046)	−0.263 (0.254)
WO		−0.260*** ^*H*3.a^ (0.037)	−0.065* (0.033)	−0.338* (0.146)		−0.187^**^^H3.b^ (0.060)	0.062 (0.059)	−0.280 (0.267)
RA		−0.203*** ^*H*4.a^ (0.045)	0.014 (0.039)	0.022 (0.146)		−0.317*** ^*H*4.b^ (0.073)	−0.047 (0.070)	−0.136 (0.266)
JI		−0.226*** ^*H*5.a^ (0.039)	−0.071* (0.033)	−0.064 (0.125)		−0.367*** ^*H*5.b^ (0.063)	−0.175** (0.059)	−0.040 (0.228)
SSE			0.366*** ^*H*6.a^ (0.046)	−0.041 (0.200)			0.419*** ^*H*6.b^ (0.083)	−0.110 (0.363)
CSE			0.256*** ^*H*7.a^ (0.030)	0.286* (0.140)			0.345*** ^*H*7.b^ (0.054)	0.320 (0.254)
Interaction terms								
WHC*SSE				−0.042* ^*H*1.c^ (0.021)				**−0.060 ^*H*1.d^ (0.039)**
WHC*CSE				**0.029 ^*H*1.e^ (0.016)**				0.061* ^*H*1.f^ (0.029)
IP*SSE				**0.021 ^*H*2.c^ (0.033)**				**0.060 ^*H*2.d^ (0.059)**
IP*CSE				**0.007 ^*H*2.e^ (0.023)**				**−0.011 ^*H*2.f^ (0.041)**
WO*SSE				0.114*** ^*H*3.c^ (0.032)				**0.097 ^*H*3.d^ (0.057)**
WO*CSE				−0.051^**^^H3.e^ (0.019)				**−0.030 ^*H*3.f^ (0.035)**
RA*SSE				**0.025 ^*H*4.c^ (0.040)**				**0.094 ^*H*4.d^ (0.072)**
RA*CSE				**−0.020 ^*H*4.e^ (0.025)**				**−0.061 ^*H*4.f^ (0.046)**
JI*SSE				**−0.038 ^*H*5.c^ (0.033)**				**−0.097 ^*H*5.d^ (0.061)**
JI*CSE				**0.031 ^*H*5.e^ (0.021)**				**0.058 ^*H*5.f^ (0.038)**
Model fit								
σ^2^	2.032***	0.691***	0.452***	0.420***	3.880***	1.827***	1.420***	1.359***
τ	0.239*	0.097*	0.046*	0.047*	0.499*	0.278**	0.245**	0.249**
ρ	0.105	0.123	0.092	0.100	0.114	0.132	0.147	0.155
Deviance	1380.139	974.875	805.212	779.748	1628.140	1346.39	1254.271	1239.099
ΔDeviance		−405.564***	−169.663***	−25.464***		−281.750***	−92.119***	−15.172***
Pseudo *R*^2^		0.660	0.346	0.071		0.529	0.223	0.043

N = 57 (supervisors); N = 380 (employees); the regression coefficients are the unstandardized coefficients from HLM; values in parentheses display the standard error from HLM; *p < 0.05. **p < 0.01. ***p < 0.001 (two-tailed); σ^2^, variance within groups ($\sigma_{w}^{2}$); τ, variance between groups ($\sigma_{B}^{2}$); ρ, intra-class correlation coefficient (ICC); deviance, −2 × log-likelihood of the full maximum-likelihood estimate (is a measure of model fit; the smaller it is, the better the model fits). WHC, work-home conflict; IP, invasion of privacy; WO, work overload; RA, role ambiguity; JI, job insecurity; SSE, social self-efficacy; CSE, computer self-efficacy; IRP, in-role performance; ERP, extra-role performance. The bolded values refer to the results of examining the hypotheses proposed.

As Table 3 exhibits, predictors were added in Model 2. The results indicated that WHC has significant negative effects on IRP and ERP (Model 2: IRP: γ = −0.125, *p* < 0.001; ERP: γ = −0.105, *p* < 0.05), providing support for hypotheses H1a and H1b, respectively. IP has not any significant effects on IRP and ERP (Model 2: IRP: γ = −0.035, *p* > 0.05; ERP: γ = −0.049, *p* > 0.05). Therefore, hypotheses H2a and H2b were both rejected. WO has significant negative effects on IRP and ERP (Model 2: IRP: γ = −0.260, *p* < 0.001; ERP: γ = −0.187, *p* < 0.01), providing support for hypotheses H3a and H3b, respectively. RA has significant negative effects on IRP and ERP (Model 2: IRP: γ = −0.203, *p* < 0.001; ERP: γ = −0.317, *p* < 0.001), providing support for hypotheses H4a and H4b, respectively. JI has significant negative effects on IRP and ERP (Model 2: IRP: γ = −0.226, *p* < 0.001; ERP: γ = −0.367, *p* < 0.001), providing support for hypotheses H5a and H5b, respectively.

In Model 3, the two moderating variables were added. The results indicated that SSE has significant positive effects on IRP and ERP (Model 3: IRP: γ = 0.366, *p* < 0.001; ERP: γ = 0.419, *p* < 0.001), providing support for hypotheses H6a and H16b, respectively. CSE has significant positive effects on IRP and ERP (Model 3: IRP: γ = 0.256, *p* < 0.001; ERP: γ = 0.345, *p* < 0.001), providing support for hypotheses H7a and H17b, respectively.

**FIGURE 1:**
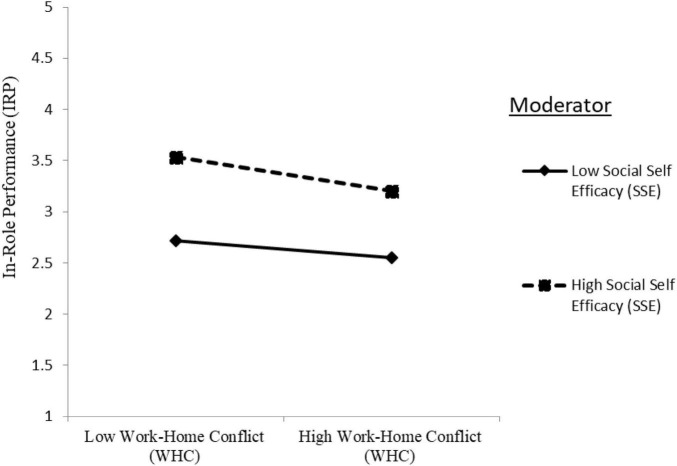
Moderation effect of social self-efficacy (SSE) on the relationship between work–home conflict (WHC) and in-role performance (IRP).

In Model 4, the interaction terms were added. The two-way interaction term of SSE with WHC in predicting IRP was significantly negative (IRP: Model 4: γ = −0.042, *p* < 0.05), providing support for hypothesis H1c. The plotted interaction in Figure 1 unveiled that WHC decreased IRP to a higher degree when SSE was high than low, indicating that SSE strengthens the negative relationship between WHC and IRP.

**FIGURE 2:**
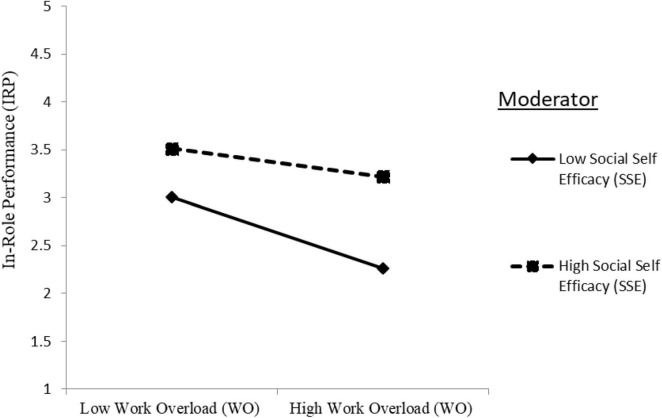
Moderation effect of social self-efficacy (SSE) on the relationship between work overload (WO) and in-role performance (IRP).

The two-way interaction term of SSE with WO in predicting the IRP was significantly positive (IRP: Model 4: γ = 0.114, *p* < 0.001), providing support for hypothesis H3c. The plotted interaction in Figure 2 unveiled that WO decreased the IRP to a higher degree when SSE was low than high, indicating SSE dampens the negative relationship between WO and IRP.

**FIGURE 3:**
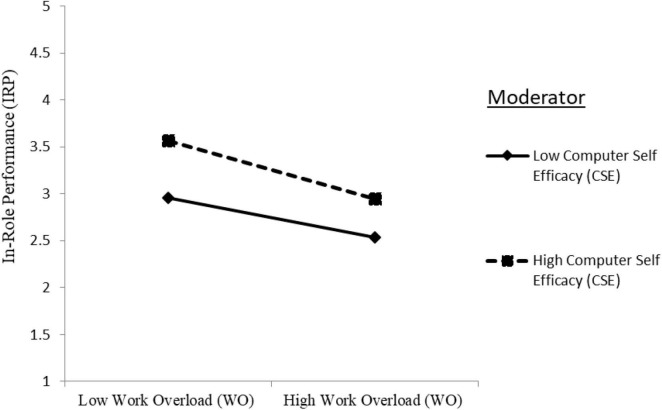
Moderation effect of computer self-efficacy (CSE) on the relationship between work overload (WO) and in-role performance (IRP).

The two-way interaction term of CSE with WO in predicting the IRP was significantly negative (IRP: Model 4: γ = −0.051, *p* < 0.01) providing support for the hypothesis H3e. The plotted interaction in Figure 3 unveiled that WO decreased IRP to a higher degree when CSE was high than low, indicating that CSE strengthens the negative relationship between WO and IRP.

**FIGURE 4:**
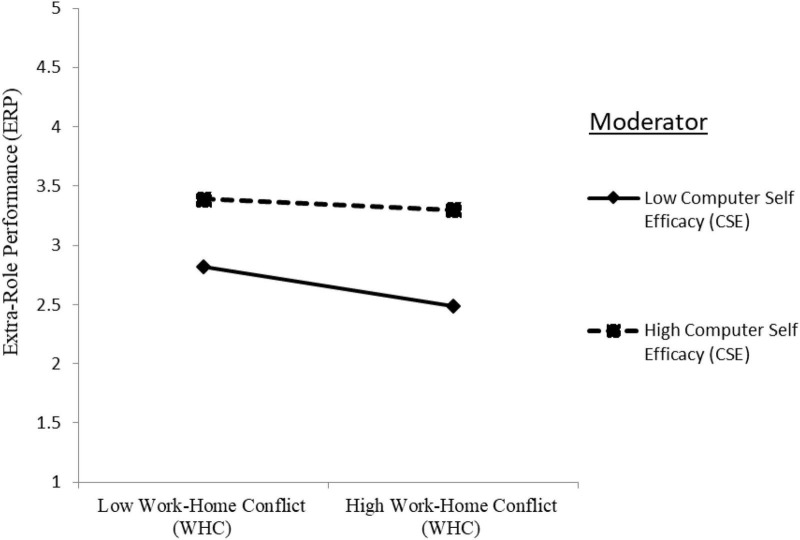
Moderation effect of computer self-efficacy (CSE) on the relationship between work–home conflict (WHC) and extra-role performance (ERP).

The two-way interaction term of CSE with WHC in predicting ERP was significantly positive (ERP: Model 4: γ = 0.061, *p* < 0.05), providing support for hypothesis H1f. The plotted interaction in Figure 4 unveiled that WHC decreased the ERP to a higher degree when CSE was low than high, indicating CSE dampens the negative relationship between WHC and ERP.

**FIGURE 5:**
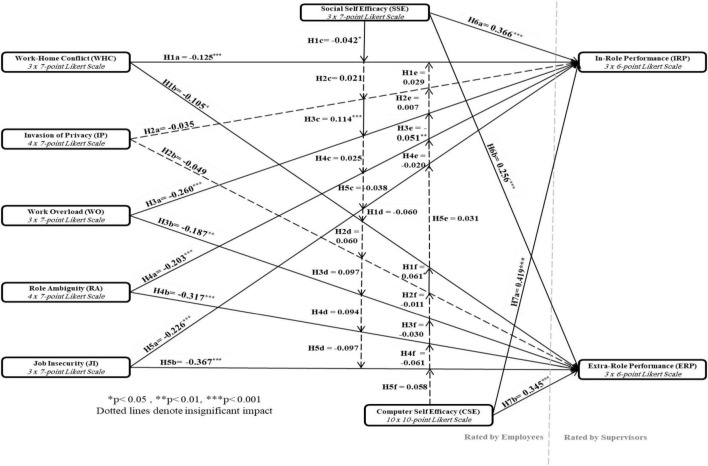
Model of findings and estimation results.

The interaction terms of SSE and CSE with the other predictors on IRP and ERP were not found as statistically significant. Therefore, hypotheses H1d, H1e, H2c, H2d, H2e, H2f, H3d, H3f, H4c, H4d, H4e, H4f, H5c, H5d, H5e, and H5f were rejected. Figure 5 represents the model of findings and the results of examining research hypotheses and finally, Appendix C presents SMART-PLS3 CFA Graphs.

## Discussion and Conclusion

The present study provides input into two research realms. In the information system research field, this study presents a conceptual and empirical validation of the idea of technostress among frontliners and examines its relationships with individual outcomes. In the organizational behavior research field, the study adds to the TMSC by recognizing and validating various types of stressors associated with the use of ICTs and by identifying possible mitigating dynamic personality differences.

Results indicate that four of the five TSCs, namely, WHC, WO, RA, and JI influence negatively IRP and ERP. Nevertheless, there is no significant relationship between the technostress IP and both IRP and ERP. A possible explanation for such finding is that theoretically distinguishing between stressors is imperative to understand the diverse relationships among stressors, strains, and other outcomes (Webster et al., 2011), although previous studies have demonstrated the negative impact of the five TSCs, when considered as one aggregated variable, on different outcomes (Ayyagari et al., 2011; Tarafdar et al., 2015).

Results also indicate that domain-linked self-efficacy beliefs, namely, SSE and CSE influence positively both IRP and ERP. Drawing on social cognitive theory (Bandura, 1997), such findings confirm that self-efficacy beliefs affect individual’s feeling of competency and confidence in his perceived skills to perform a task.

This study sought to extend the TMSC. It endeavors to understand why and how different TSCs affect different outcomes through different self-efficacy beliefs. Evaluating the moderating relations proposed in H1c:H5c; in H1d:H5d, in H1e:H5e, and in H1e:H5e reveals that SSE strengthens the negative relationship between WHC and IRP, and dampens the relationship between WO and IRP. Concerning CSE, results indicate that CSE strengthens the relationship between WO and IRP and dampens the relationship between WHC and ERP. Conferring to Bandura’s (1997) theory, the outcomes of self-efficacy belief generally fall into four main categories. First, SE influences the activities and situations that affect an individual’s selected behavior. Second, SE influences the extent to which an individual will employ the necessary effort to overcome obstacles and persevere when confronted with adverse circumstances. Third, SE influences an individual’s feelings of anxiety and stress. Fourth, SE predicts an individual’s performance and coping behavior (Tu et al., 2005). Moreover, the predictive capability of SE is stronger and more accurate when determined by specific domain-linked measures rather than with general measures (Saleem et al., 2011). Therefore, the level of an individual’s domain-linked SE influences differently an employee’s perception of exercising control over each TSC on his or her performance.

Conversely to expectations, findings indicate that the effect of the five TSCs on ERP is almost constant across all the levels of SSE (H1d:H5d) and that there is no moderating effect of SSE in the relationships between JI (H5c), IP (H2c), RA (H4c), and IRP. Besides, there is no moderating effect of CSE in the relationships between WHC (H1e), IP (H2e), RA (H4e), JI (H5e), and IRP. In addition, there is no moderating effect of CSE in the relationships between WHC (H1f), IP (H2f), RA (H4f), JI (H5f), and ERP.

A plausible reason for the non-support of the abovementioned hypotheses could be that no objective criterion is adequate to describe a situation as stressful and that only the individual experiencing the event can do so (Lazarus and Folkman, 1984). Furthermore, the fact that an individual’s behavior varies from situation to situation, may not necessarily mean that behavior is controlled by the situation but rather that the person is construing the situation differently and consequently the same set of stimuli may provoke diverse responses from different persons or from the same person at different times (Jones, 1989). These findings raise the question about the possible role of personality differences, other than SE beliefs, in influencing employee’s TSCs–outcomes relationships.

Although the majority of the moderating hypotheses were not supported, a holistic interpretation of the research results provides two major insights into the business literature: first, the relationship between a specific TSC and a specific outcome is not similarly influenced by different domain-linked SE. Second, the relationships between each of the five TSCs and a specific outcome are not similarly moderated by the same domain-linked SE.

Finally, as the literature lacks related studies, the validation of the result could not be established. It is hoped that the findings of this research will provide an avenue for academic research to address technostress effects and inhibitors in different environments.

### Theoretical Implications

This study makes vital contributions to the technostress research. First, former literature concerned with organizational stress issues has established the substantial relation of work stressors with undesirable job outcomes. In the same view, the technostress literature has investigated the prominent role of TSCs in generating disagreeable outcomes like an increase in employee strain and a decrease in employee job satisfaction and productivity (Ragu-Nathan et al., 2008; Ayyagari et al., 2011).

Nevertheless, an outstanding discourse about occupational stress perceives stress as a normal repercussion of living and theorizes it as the normal behavioral response to external stimulis (Richmond and Kehoe, 1999). Furthermore, scientists such as Selye (1987) have discerned between distress and eustress, in other words between bad stress and good stress. This category of stress research was grounded on different models of occupational stress and found that distress engenders negative job outcomes while eustress engenders positive job outcomes (Selye, 1987; Le Fevre et al., 2003). Although the organizational literature has underlined the prominent concept of good and bad stress, the technostress literature has not yet. Given that the positive aspects of TSCs and their possible positive outcomes have not been broadly leveraged in the technostress literature (Srivastava et al., 2015), the current study is one of the first to theorize and empirically establish that different TSCs in some situations may produce positive outcomes. In addition, the findings that the same TSC can be perceived as either controllable or not by individuals with different efficacy beliefs extend the stream of current studies that investigate the possible mitigating effect of organizational-level approaches (Tarafdar et al., 2015; Krishnan, 2017) and static personality-level approaches (Khedhaouria and Cucchi, 2019), directed in different contexts to manage technostress. Hence, the current research extends the understanding of the mechanisms through which different TSCs lead to different outcomes and shifts the focus from the negative aspects of TSCs and outcomes to both positive and negative aspects.

Second, this study incorporates the substantial role of personality into the technostress literature. The current research is grounded in the TMSC, it combines the significant role of personality differences with the technostress–job outcomes model, theorizes and empirically examines the moderating impact of self-efficacy on the relationships between each TSC and job outcomes. This is particularly interesting since the coping mechanisms adopted by individuals with different self-efficacy beliefs are quite different, causing differences in their perception of their ability to exercise control over each TSC leading to differences in the resulting work outcomes. Hereafter, one of the main contributions of this research is founding the prominence of dynamic personality differences for defining the impact of each TSC on employee’s job outcomes. Indeed, including personality characteristics into well-established information system research models was found to significantly boost their predictive power (Devaraj et al., 2008; Junglas et al., 2008). This study deepens the stress theories’ context by explicitly integrating the coping concept into the technostress phenomena, provides a holistic understanding of the technostress process from an IS–social interaction perspective and combines self-efficacy with the TMSC which results in a more robust theoretical model with better explanatory power than an isolated examination of the model’s facets could render.

Third, although prior research has tested different aspects of IT characteristics, tasks, and users as possible interventions that buffer the impacts of TSC on various outcomes (Krishnan, 2017; Khedhaouria and Cucchi, 2019), the potential moderating role of domain-linked SE in the relation between TSC and FLE’s outcomes, has been predominantly ignored. This study incorporated two domain-linked SEs and is thus consistent with Marakas et al.’s (1998) study, which used domain-specific SE leading to the most credible research findings regarding SE. Furthermore, it provides an in-depth understanding of the role of CSE in the technostress phenomenon by suggesting that this role is more complicated than the simple, direct impact of CSE on TSC would suggest. Besides, the call for additional research about SSE in organizational settings is ongoing (Luo et al., 2019). This research is one of few to introduce SSE within the technostress literature and adds to the emerging evidence that SSE is a highly relevant variable in organizational settings. Indeed, this study is the first to conceptualize the moderating effects of CSE and SSE in the relationship between each type of TSCs and in-role and extra-role performances.

Fourth, this study is one of the first to conceptualize each dimension of TSC’s impact on job outcomes and concluded that TSCs are not identical and do not lead to identical outcomes. Consistent with Webster et al. (2011), findings demonstrate that making a theoretical distinction between stressors is imperative for understanding the different relationships among stressors, strains, and other important outcomes.

Fifth, although there has been some doubt about the capacity of SE to influence performance within a complicated job setting (Krishnan et al., 2013), this study’s findings propose that, even in a complicated job situation, the perceptions of domain-linked SE are correlated with FLE’s performance, especially after discerning between in-role and extra-role performances.

Sixth, since the supervisor rating of an employee’s performance is more predictive of outcomes than the employee rating of his/her performance (Atkins and Wood, 2006), this study is one of the few to confirm that supervisor rating would allow for an enhanced understanding of the performance paradigm and its antecedents, especially when using HLM statistical approach.

Finally, this research clearly distinguishes between in-role and extra-role performances and is consistent with MacKenzie et al.’s (1998) findings implying that they are both theorized to have dissimilar antecedents.

### Managerial Implications

First, this study can be considered a pioneer study for understanding how the dynamic personality differences of FLEs using ICT for work purposes affect job outcomes. While the personality static traits are difficult to change, the dynamic personality differences undergo substantial internal transformation throughout a story. Self-efficacy beliefs are modifiable protective factors (Shoji et al., 2016) theorized in this research as a form of coping mechanism and coping strategies are not mutually exclusive. Indeed, individuals differ in their choices of coping strategies and in the degree to which they engage in a definite strategy (Hauk et al., 2019). Thus, identifying the possible role that self-efficacy might play in influencing FLE’s job outcomes as negative or positive can be leveraged by ICT designers and managers in several ways.

Second, the research findings prove that it is not appropriate to assume a “one-size-fits-all approach” in ICT usage and ICT-related technostress for FLEs. Organizational management can wisely propose and implement ICT usage rules to manage and control ICT-induced technostress for FLEs with different self-efficacy beliefs.

Keeping personality differences in mind while describing job potentials might raise FLE’s positive job outcomes and alleviate their negative job outcomes. For instance, results indicate that FLEs with high levels of CSE beliefs might be assigned to complex loaded ICT-related tasks as they will react to such assignments with a feeling of controllability, perceive it positively, and work on it to ameliorate their in-role job performance. Besides, management can assign after-work tasks to individuals with high CSE who do not perceive such assignments negatively and consequently exert high extra-role performance without suffering from WHC. Management can also assign excessive workload or after-work tasks for FLEs with high SSE beliefs who can perceive such assignments positively, exert an ability in multitasking and accordingly overcome the exposure to task-related ICT leading to better IRP.

Hence, findings from this study concerning the relationships between TSCs and self-efficacy beliefs have implications for managing the influence of personality dynamic differences on TSCs and related outcomes.

Third, this study’s findings suggest that self-efficacy influences the nature of technostress experienced by FLEs and their response to each TSC. It strengthens the role of personality characteristics within the organizational behavior stress literature, suggesting that diverse self-efficacy beliefs help FLEs cope differently with different TSCs. Understanding employees’ stress-related personalities can support an organization in developing better stress-management strategies. For instance, organizations can support their employees develop improved coping skills such as promoting a take-charge attitude toward a problem or employing social support or a definite control strategy to deal prudently with a stressful situation (Carver et al., 1989). Inside the organization, strategies to detect, manage, and inhibit TSCs can be arranged at multiple levels. For example, the individual-focused strategies can embrace managing mechanisms to cope with TSCs or amending responses to foreseeable ICT-related difficulties. In addition, the organization-focused strategy might embrace adjusting the physical task demands or the interpersonal work demands. Such strategies can be formulated by the management based on essential differences in domain-linked efficacy beliefs of particular FLEs.

Furthermore, Bandura (1977), suggests that several treatment techniques supply one or more of the four sources of self-efficacy information are consequently successful since they yield change by raising individual’s perceived self-efficacy. Thus, combining the required formulated strategy with the necessary self-efficacy’s treatment techniques allows management to maintain an adequate employee’s self-efficacy level. Such practice enhances employees’ beliefs that TSC is surmountable, that overcoming TSC leads to better achievements, enhances technology to be a solution and not a problem, and helps an organization make resource allocation decisions toward positively oriented job outcomes.

Finally, organizations should consider that various “problems” usually classified as “work stress” may be symptoms of possibly non-related organizational issues, an accurate organizational diagnosis and evaluation must precede any stress management intervention.

### Limitations and Future Research

This study has a few limitations. First, the SE measure’s responses were based on using a hypothetical scenario (Compeau and Higgins, 1995) and raised an issue about the respondents’ accountability to represent real-world situations. To counter this limitation, and as performed by Compeau and Higgins (1995), employees who answered the pilot study were asked before filling the questionnaire if the hypothetical scenario can represent a real situation in their quotidian life and if they are capable to imagine what is requested from them to answer the items. They all agreed that this is not difficult.

Second, recent literature has focused on examining TSCs’ antecedents and inhibitors and their impact on technology users’ outcomes. It will be interesting for future research to consider their impact on clientele’s outcomes. Besides, the analysis of SSE and CSE moderating effects emphasized the need for future research to integrate static and dynamic constructs of personality differences and investigates their potential effects on the TSCs–outcomes relationships. A supplementary avenue for additional research could be to propose organizational strategies that adequately manage relevant technostress. In this view, careful consideration of personality differences is crucial in the implementation of organizational learning strategies since knowledge management systems and technologies have become more dominant in a business corporation.

Third, this study’s constructs are promising foundations for investigating FLEs’ behavior when facing TSCs. It would be interesting to examine the same research model in different service settings to better understand the differences and similarities between FLE efficacy beliefs in terms of the manifestation of job outcomes related to TSC.

Fourth, human-to-human interaction (HHI) is considered a new challenging research realm. The social role of computer-mediated communication, for HHI, for which human–computer interaction represents the foundation, is particularly evident when individuals need to interconnect with information provision systems, or among them, to acquire information or to share them securely (Boca et al., 2013). This study examined the effect of technostress on FLE’s HHI role that involves human–computer interaction and overlaps with computer-mediated communication. Nonetheless, researchers could explore the differences between the effect of TSCs due to computer-mediated communication as compared with those due to human–computer interaction on FLE’s outcomes. Such understanding can assist both the technostress and the HHI literature in making a careful choice of the most suitable treatment techniques for FLE’s stress relief.

Fifth, as this main research aim was to emphasize the importance of individual dynamic differences in moderating the relationships between TSCs and outcomes, and since adding control variables to this research model would make it more complicated and increase the risk of inter-covariance problems, the control variables were not added to the model to be tested. These variables themselves may pose interesting research questions in terms of how such factors play into the relationship between each of the five TSCs and various outcomes in a definite setting.

To end, this study will be supportive in notifying other facets of the harmful aspects of the digital age in forthcoming research. For instance, self-efficacy beliefs can be influential to additional hurtful processes far off technostress such as cyberbullying and techno-addiction. Certainly, the relationships tested in this research can be used for supplementary advanced examination in this vital area.

## Conclusion

Despite these limitations, this present research provides an essay about the importance of dynamic personality differences as coping factors that influence diverse outcomes correlated to technostress in non-western literature and closes significant research gaps.

Finally, the world is witnessing the repercussions of the COVID-19 pandemic, which is convulsing individuals’ lives and necessitating reliable telecommunicating infrastructure and an individual’s vital characteristics that shape his or her capacities to adapt.

Although working remotely is not a new phenomenon for ICT frontliners, the extensity due to the COVID-19 pandemic is novel. This research provides an initial impetus for upcoming studies about CSE as a crucial individual characteristic that helps people adapt to the technological widespread and sheds light on SSE as a necessary characteristic required for managing social interactions when life returns to a post-pandemic new normal.

## Data Availability Statement

The raw data supporting the conclusions of this article will be made available by the authors, without undue reservation.

## Ethics Statement

Ethical review and approval was not required for the study on human participants in accordance with the local legislation and institutional requirements. Written informed consent for participation was not required for this study in accordance with the national legislation and the institutional requirements.

## Author Contributions

RS prepared the data analysis section and results. JS wrote the rest of the manuscript and collected the data. ZG revised and upgraded the manuscript. All authors contributed to the article and approved the submitted version.

## Conflict of Interest

The authors declare that the research was conducted in the absence of any commercial or financial relationships that could be construed as a potential conflict of interest.

## Publisher’s Note

All claims expressed in this article are solely those of the authors and do not necessarily represent those of their affiliated organizations, or those of the publisher, the editors and the reviewers. Any product that may be evaluated in this article, or claim that may be made by its manufacturer, is not guaranteed or endorsed by the publisher.

## Figures and Tables

**FIGURE A1:**
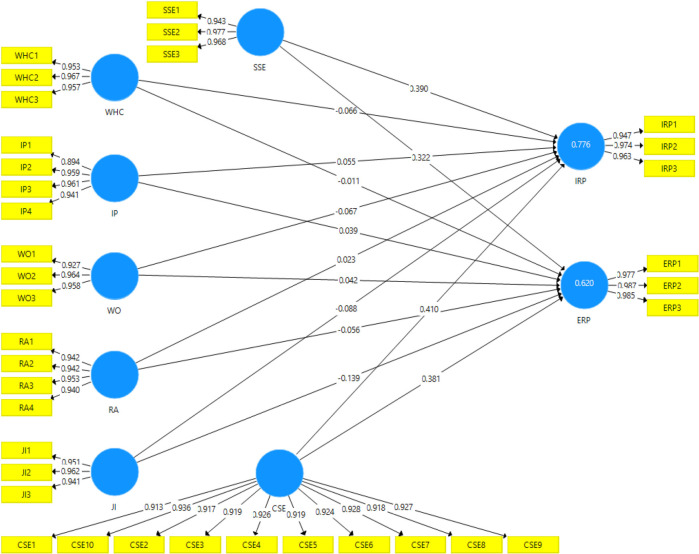
Measurement Model using SMART-PLS3.

## APPENDIX A

**TABLE A1:** Questionnaire and corresponding codifications.

WHC1	Using ICTs blurs boundaries between my job and my home life.
WHC2	Using ICTs for work-related responsibilities creates conflicts with my home responsibilities.
WHC3	I do not get everything done at home because I find myself completing job-related work due to ICTs.
IP1	I feel uncomfortable that my use of ICTs can be easily monitored.
IP2	I feel my privacy can be compromised because my activities using ICTs can be traced.
IP3	I feel my employer could violate my privacy by tracking my activities using ICTs.
IP4	I feel that my use of ICTs makes it easier to invade my privacy.
WO1	ICTs create many more requests, problems, or complaints in my job than I would otherwise experience.
WO2	I feel busy or rushed due to ICTs.
WO3	I feel pressured due to ICTs.
RA1	I am unsure whether I have to deal with ICT problems or with my work activities.
RA2	I am unsure what to prioritize: dealing with ICT problems or my work activities.
RA3	I can NOT allocate time properly for my work activities because my time spent on ICT activities varies.
RA4	Time spent resolving ICT problems takes time away from fulfilling my work responsibilities.
JI1	ICTs will advance to an extent where my present job can be performed by a less skilled individual.
JI2	I am worried that new ICTs may pose a threat to my job.
JI3	I believe that ICTs make it easier for other people to perform my work activities.
SSE1	I can understand other people’s feeling.
SSE2	I can predict how others will react to my behavior.
SSE3	I can anticipate others’ reactions to what I do.
Often in our jobs we are told about software packages that are available to make work easier. For the following questions, imagine that you were given a new software package for some aspect of your work. The following questions ask you to indicate whether you could use this unfamiliar software package under a variety of conditions.	
CSE1	If there was no one around to tell me what to do as I go.
CSE2	If I had never used a system like it before
CSE3	If I had only the instructions for reference.
CSE4	If I had seen someone else using it before trying it myself.
CSE5	If I could call someone for help if I got stuck.
CSE6	If someone else had helped me get started.
CSE7	If I had a lot of time to complete the job for which the system was provided.
CSE8	If I had just the built-in help facility for assistance.
CSE9	If someone showed me how to do it rest.
CSE10	If I had used a similar system before this one to do the same job
“Within the last 6 months how often did this employee…”	
IRP1	Meet formal performance requirements when serving customers?
IRP2	Perform all those tasks for customers that were required of him/her?
IRP3	Adequately complete all expected customer service behaviors?
ERP1	Go above and beyond the “call of duty” when serving customers?
ERP2	Willingly go out of his/her way to make a customer satisfied?
ERP3	Help customers with problems beyond what was expected or required?

## Appendix B

**TABLE A2:** Respondent’s profile.

Category	Frequency	Percentage
**Gender**		
Male	246	65
Female	134	35
**Annual income in USD**		
Below 24,000	152	40
24,000–48,000	130	34
48,000–72,000	83	22
Above 72,000	15	4
**Education**		
Primary school	23	6
Bachelor’s degree	182	48
Master’s degree	160	42
Doctoral degree	15	4
**Age**		
Between 18 and 25 years old	83	22
Between 26 and 35 years old	172	45
Above 35 years old	125	33
**Work experience**		
Less than 5 years	121	32
Between 5 and 10 years	126	33
Between 11 and 15 years	72	19
More than 15 years	61	16
**Family status**		
Single	209	55
Married	155	41
Divorced	16	4

## Abbreviations

CSE: computer self-efficacy
CFA: confirmatory factor analysis
ERP: extra-role job performance
ICT: information and communication technology
IRP: in-role job performance
IP: invasion of privacy
JI: job insecurity
RA: role ambiguity
SE: self-efficacy
SSE: social self-efficacy
TSCs: technostress creators
TMSC: transactional model of stress and coping
WHC: work–home conflict
WO: work overload.
